# Quality of Virtual vs. In-Person Outpatient Palliative Care: Disparities by Language and Race

**DOI:** 10.1016/j.jpainsymman.2024.12.016

**Published:** 2025-01-03

**Authors:** Sarah Nouri, Steven Z. Pantilat, Christine S. Ritchie, Courtney R. Lyles, Ying Shi, David O’Riordan, John Boscardin, Rebecca L. Sudore

**Affiliations:** Division of Palliative Medicine, University of California San Francisco, San Francisco, California, USA; Department of Medicine, University of California San Francisco, San Francisco, California, USA; Division of Palliative Medicine, University of California San Francisco, San Francisco, California, USA; Department of Medicine, University of California San Francisco, San Francisco, California, USA; Center for Aging and Serious Illness, Division of Palliative Care and Geriatric Medicine, Department of Medicine, Massachusetts General Hospital and Harvard Medical School, Boston, Massachusetts, USA; Center for Healthcare Policy and Research, Department of Public Health Sciences, University of California Davis, Sacramento, California, USA; Division of Geriatrics, University of California San Francisco, San Francisco, California, USA; Department of Medicine, University of California San Francisco, San Francisco, California, USA; Division of Palliative Medicine, University of California San Francisco, San Francisco, California, USA; Department of Medicine, University of California San Francisco, San Francisco, California, USA; Division of Geriatrics, University of California San Francisco, San Francisco, California, USA; Department of Medicine, University of California San Francisco, San Francisco, California, USA; Department of Epidemiology & Biostatistics, University of California San Francisco, San Francisco, California, USA; Division of Geriatrics, University of California San Francisco, San Francisco, California, USA; Department of Medicine, University of California San Francisco, San Francisco, California, USA

**Keywords:** Outpatient palliative care, Health disparities, Limited English proficiency, Outcomes

## Abstract

**Context.:**

Virtual visits have increased in outpatient, clinic-based palliative care (OPC). The association between virtual visits and OPC outcomes is largely unknown.

**Objectives.:**

(1) Examine the association between visit type (virtual vs. in-person) and screening (yes/no) for psychosocial, spiritual, and goals of care needs. (2) Assess effect modification by language.

**Methods.:**

We used data from the Palliative Care Quality Network (01/2017–03/2021). We conducted multivariable analyses adjusting for age, sex, diagnosis, self-reported race-ethnicity, and language, clustered by site, and included an interaction term to assess effect modification by language.

**Results.:**

Among 2684 patients, 29% had a virtual visit; 50% were ≥65 years old, 24% non-English preferred languages; 18% identified as Hispanic, 9% Black, 17% Asian, 6% Native Hawaiian/Pacific Islander. There were no differences by visit type in screening for psychosocial (aOR 0.87 vs. in-person visits, 95% CI 0.60–1.25), spiritual (aOR 0.81, 95% CI 0.57–1.15), or goals of care needs (aOR 1.05, 95% CI 0.85–1.31). Patients with non-English preferred languages (vs. English-speaking) had significantly lower odds of screening regardless of visit type. Patients identifying as Black (vs. White) also had significantly lower odds of screening.

**Conclusions.:**

There were no differences by visit type in screening for psychosocial, spiritual, and goals of care needs. Patients with preferred languages other than English were significantly less likely to be screened than English speakers, though there was no further difference by visit type. Patients identifying as Black were also significantly less likely to be screened. Addressing these disparities in core OPC elements is essential in providing equitable, high-quality OPC.

## Introduction

Patient-clinician video and telephone visits, hereafter referred to as virtual visits, have increased rapidly over the past decade in outpatient, clinic-based palliative care.^1-10^ Since the coronavirus pandemic, virtual visits have become a norm, and with an increasing number of homebound older adults with serious illness, virtual visits represent a promising opportunity for increasing access to outpatient palliative care.^1,9-11^ However, the association between virtual visits and palliative care outcomes is largely unknown, particularly among marginalized populations experiencing systemic patterns of disadvantage, including the >25 million people in the US with preferred languages other than English.^12^

While different patient subgroups engage in both palliative care and virtual visits differently, there have been no studies to our knowledge that have examined differences in palliative care visits by visit modality and patient characteristics simultaneously.^9,13-15^ This gap in knowledge about the intersection between virtual visits and palliative care quality may lead to worsening disparities among people with non-English preferred languages. Moreover, little is known about the quality of palliative care among this population. A recent study found poorer quality of patient-oncologist communication among patients with non-English preferred languages, particularly regarding psychosocial topics and despite the use of professional interpreters.^16^ Screening for psychosocial, spiritual, and goals of care needs is a core element of outpatient palliative care.^17^ Because communication cues may be different in a virtual format compared to an in-person visit, understanding the association between these palliative care screening outcomes and virtual visits is critical, particularly among those with non-English preferred languages. In this study, we used multisite data from the Palliative Care Quality Network (PCQN) to examine the association between virtual and in-person outpatient palliative care visits and screening for psychosocial, spiritual, and goals of care needs and to examine possible effect modification of language preference on visit type.

## Methods

### Study Sample

We report methods according to STROBE guidelines for cross-sectional studies. We used data from the PCQN, a national, multisite collaborative of interdisciplinary palliative care teams that collect standardized patient data.^18^ Data are all collected as part of usual patient care—either recorded by clinicians during visits or by patients in previsit surveys. Fifty-eight outpatient palliative care teams (referred to as “sites”), including both academic and community programs in 11 states, participate in PCQN. Because of our focus on marginalized populations, we restricted the cohort to patients from sites that consistently recorded race/ethnicity and preferred language data (i.e., <10% missing; 45 programs were excluded) and saw ≥50 unique patients during the study period (7 programs were excluded). This decision resulted in a cohort of six PCQN sites all located in California and Hawai’i. We included all adults (18+ years old) with at least one outpatient palliative care visit at the included sites between January 2017 and March 2021. This study was approved by the University of California San Francisco Institutional Review Board (IRB #16-18596).

### Measures

Dichotomous outcome measures were: 1) screening for psychosocial needs, 2) screening for spiritual needs, and 3) screening for goals of care needs at patients’ initial outpatient palliative care visit. Screening for psychosocial needs could include assessing emotional status (e.g., discussing mood, coping strategies), managing psychological symptoms or diagnoses, or assessing psychosocial context (e.g., discussing caregiver support).^19^ Screening for spiritual needs could include assessing faith-based, religious, or meaning-making practices or managing existential distress. Screening for goals of care needs could include establishing illness understanding, supporting decision-making (in-the-moment or future), or end-of-life planning. Clinicians reported outcome measures in their notes using structured fields “yes” or “no” to indicate whether they did or did not screen the patient for psychosocial needs, spiritual needs, or goals of care needs.

The primary predictor measure was visit type, defined as either in-person or virtual. Covariates demonstrated to impact communication per the Structural Influence Model^20^ included age, sex (male or female), self-reported race-ethnicity (non-Hispanic White, Hispanic/Latinx, Black/African American, Asian, Other including Native Hawaiian/Pacific Islander, American Indian/Alaska Native, and all other races and/or ethnicities not included in other categories), and preferred language (English, Spanish, Chinese, and Other), abstracted from core electronic health record data at each site, as well as primary diagnosis and reason for referral to palliative care, both of which are categorical variables standardized in clinician notes across all PCQN sites.

### Statistical Analyses

We used descriptive statistics to report all variables and t-tests, Chi-squared tests, or Fisher’s exact tests for unadjusted analyses. To examine the adjusted association between visit type and each outcome variable, we used multivariable logistic regression adjusting for age, sex, primary diagnosis, and race-ethnicity, accounting for intrasite correlations through cluster-robust standard errors.^21^ We checked for multicollinearity of predictor variables using the variance inflation factor metric. To determine effect modification by preferred language, we included an interaction of preferred language and visit type. Participants with missing data (3%) were omitted from multivariable analysis. Due to the sample size, we dichotomized preferred language to English vs. Other. To assess the possible impact of COVID-related telehealth implementation, we repeated all analyses, adjusting for language and the interaction of year (2017–2019 vs. 2020–2021) by visit type.

## Results

### Participant and Site Characteristics

Of the six included sites, three were public safety-net hospitals, two were academic hospitals, and one was a non-profit community hospital. Outpatient palliative care teams at all sites had physicians, nurses or nurse practitioners, and social workers; one site did not have a chaplain.

During the study period, 2,684 adult patients had an initial outpatient palliative care visit at one of these six sites. Overall, 50% were ≥65 years old; 47% identified as White, 18% Hispanic, 17% Asian, 9% Black, 6% Native Hawaiian or Pacific Islander; 11% were Spanish-speaking, 6% Chinese-speaking (Cantonese, Mandarin, or other), and 6% had another non-English language preference (Table 1). In addition, 59% of patients had cancer and 15% had a neurologic illness; 52% were referred for pain, 47% for goals of care, and 36% for support.

Of 2,684 initial visits, 780 (29%) were conducted virtually. Older adults were more likely to be seen virtually, as were White and English-speaking patients. People referred for pain or with a diagnosis of cancer were more likely to be seen in person (Table 1). Prior to COVID, 25% of visits were virtual vs. 75% during COVID.

### Unadjusted Analyses

In unadjusted analyses, there were no differences by visit type in screening for psychosocial needs (75% virtual vs 73% in-person; *P* = 0.18) or spiritual needs (56% vs. 59%; *P* = 0.18; Table 2). Patients were more likely to be screened for goals of care needs in virtual visits (71%) vs. in-person visits (64%; *P* = 0.001).

Patients with a preferred language other than English were significantly less likely to be screened for psychosocial needs (65% vs. 77% with English as a preferred language; *P* < 0.001), spiritual needs (52% vs. 62%; *P* < 0.001), and goals of care needs (57% vs. 70%; *P* < 0.001). These differences in screening were most notable for Spanish-speaking and Chinese-speaking patients (Table 3). Patients identifying as Hispanic/Latinx or Black/African American were significantly less likely to be screened for psychosocial needs (66% Hispanic/Latinx and 58% Black/African American vs. 78% White/Asian/Other; *P* < 0.001), spiritual needs (53% and 45% vs. 63%; *P* < 0.001), and goals of care needs (57% and 48% vs. 78%; *P* < 0.001; Table 4).

### Adjusted Analyses

After adjusting for age, sex, primary diagnosis, race-ethnicity, and preferred language and clustering by site, there were no differences by visit type in screening for psychosocial needs (aOR 0.87, 95% CI 0.60–1.25), spiritual needs (aOR 0.81, 95% CI 0.57–1.15), or goals of care needs (aOR 1.05, 95% CI 0.85–1.31; Table 5).

Regardless of visit type, compared to patients with a preferred language of English, those with a preferred language other than English had significantly lower odds of screening for psychosocial needs (aOR 0.47, 95% CI 0.38–0.58), spiritual needs (aOR 0.54, 95% CI 0.47–0.62), or goals of care needs (aOR 0.48, 95% CI 0.39–0.59). Compared to those identifying as White, patients identifying as Black had significantly lower odds of screening for psychosocial needs (aOR 0.38, 95% CI 0.29–0.51), spiritual needs (aOR 0.51, 95% CI 0.38–0.69), or goals of care needs (aOR 0.41, 95% CI 0.34–0.49). Patients identifying as Asian had significantly higher odds of screening for goals of care needs (aOR 1.53, 95% CI 1.06–2.23).

Interaction terms in each model for language by visit type were not significant: there were no differences in screening between virtual and in-person visits for patients with preferred languages other than English compared with English-speaking patients. Interaction terms for pre-COVID vs. COVID time periods were not significant in models of screening for psychosocial needs or goals of care needs, but there was a significant interaction for the time period on screening for spiritual needs (*P* = 0.046). Patients who had an initial visit pre-COVID had significantly higher odds of being screened for spiritual needs in virtual vs. in-person visits (aOR 1.50, 95% CI 1.03–2.20), while there was no difference in screening for spiritual needs by visit type during COVID (aOR 0.71, 95% CI 0.39–1.30; Appendix).

## Discussion

This is the first study to our knowledge to examine the association of virtual visits with outpatient palliative care screening quality. Using multisite, standardized data we found that there were no differences in screening for psychosocial, spiritual, or goals of care needs in virtual vs. in-person outpatient palliative care visits, regardless of language. However, patients with preferred languages other than English had significantly lower rates of screening for psychosocial, spiritual, and goals of care needs regardless of visit type, even after adjusting for other clinical and sociodemographic characteristics.

Given virtual visits are the new norm and, in many cases, preferred by patients with serious illness, it is reassuring that there were similar rates of screening regardless of visit type.^22^ However, there were stark and concerning disparities in the quality of palliative care for patients with non-English preferred languages, specifically in screening for key, basic elements of outpatient palliative care (psychosocial, spiritual, and goals of care). In prior research, we found that patients with preferred languages other than English were less likely to follow up with our outpatient palliative care program at an urban academic center compared to those with English as a preferred language.^23^ Based on the findings in this study, it is possible that a less complete initial visit impacts patients’ desires to continue to receive palliative care services. Being less likely to follow up also means that these patients cannot be screened for these core issues during a later visit. A reason for this disparity in screening may be a lack of time.^13,24^ Interpretation adds time to visits, which can be challenging for clinicians in busy outpatient clinics where demand for services typically outstrips supply. In a study of family meetings in the ICU, clinician speech time in interpreted meetings was nearly half that of noninterpreted meetings.^24^ Limited time due to interpretation may require clinicians to prioritize urgent physical symptom needs during initial visits.^25^ With the expansion of time-based billing options in 2021, extending outpatient visits for patients requiring language interpretation may mitigate some disparities in screening. Studies in primary care, oncology, and intensive care have demonstrated that clinicians in interpreted visits are less likely to communicate about psychosocial topics, convey hope, or use active listening techniques, and are more likely to use medical jargon.^13,24,26,27^ One reason may be the potentially weaker emotional rapport between clinicians and patients in interpreted interactions, which may have led to decreased screening in this study.^26^ We could not collect information on the use of interpreters or patient-clinician language concordance in this study. However, prior research of PCQN data shows that there is a paucity of clinicians who speak a language other than English on most palliative care teams.^28^

While not an a priori focus of our study, we found that patients identifying as Black had significantly lower odds of screening for psychosocial, spiritual, and goals of care needs compared to those identifying as White. Given these screenings are led and documented by clinicians, this finding raises concern for implicit bias and anti-Black racism, which are pervasive in healthcare.^29,30^ While studies of interpersonal discrimination in serious illness care are limited, research has demonstrated that palliative care-facilitated goals of care discussions do determine end-of-life outcomes (e. g., hospice enrollment), including among patients identifying as Black, underscoring the harm of not screening for goals of care in this population.^31,32^ Despite the importance of religion and spirituality in the Black community, with spirituality often serving as a framework for medical decision-making for Black patients, screening for spiritual needs was actually lowest in this population. On further examination, we noted that the majority (66%) of Black patients were seen at a single site, suggesting local practice patterns may have impacted our findings. However, sensitivity analyses excluding that site from our models did not alter trends that patients identifying as Black had lower odds of screening for psychosocial, spiritual, or goals of care needs (data available upon request).

Spiritual screening overall was less common than for other core aspects of palliative care, with only 58% of patients being screened during their initial outpatient palliative care visit. Physicians have been shown to have very low rates of screening for spiritual needs even when caring for people with serious illness in intensive care settings (14%).^33^ Compared to these rates, in this study rates of screening for spiritual needs by palliative care clinicians in this study were higher (58%), which is expected given their additional specialty training. Nevertheless, rates of spiritual screening in this study were still lower than screening for psychosocial (73%) or goals of care needs (66%). Our analyses did not adjust for which team members were present during visits due to limitations of our data; notably, however, one of the six sites did not have a chaplain and most only had one chaplain for their entire outpatient palliative care program. It is possible that screening rates may be higher in visits that include chaplains.

This study has several limitations. We included only six outpatient palliative care programs, all of which are in California and Hawai’i, limiting the generalizability of our findings. Nevertheless, most patients identified as a minoritized race or ethnicity, and the sites included a mix of safety-net, academic, and community hospitals. Given the cross-sectional design, we could not account for all possible confounding variables, as described above (e.g., language concordance) and including other variables described in the Structural Influence Model of communication (e.g., socioeconomic status, rurality). Although the PCQN provides the largest available dataset in the US to examine palliative care outcomes, we had to group all non-English preferred languages together due to small sample sizes, losing granularity and nuance. We also did not have data on language concordance, interpreter use, or on types of interpreters used (e.g., professional vs. ad hoc, in-person vs. video vs. telephone, etc.). While we found in prior studies at UCSF that patients who speak a language other than English were less likely to follow up, it is possible that disparities in screening are mitigated over time as patients and clinicians develop rapport and have more time to address additional core issues.

In conclusion, we found reasons to be reassured regarding the quality of care provided in virtual visits. However, while we found that there are no differences by visit type in screening for psychosocial, spiritual, and goals of care needs overall and by language, we found significant disparities in screening with people with preferred languages other than English and identifying as Black being significantly less likely to be screened, regardless of visit type. Given these screenings are core elements of high-quality outpatient palliative care, it is critically important to develop and evaluate interventions to address these disparities. The disparities identified in this study by language and race also underscore the need to diversify the palliative care workforce, as research has shown the tremendous positive impact of patient-clinician racial-ethnic and linguistic concordance. Given the promise of virtual visits in expanding the reach of and access to palliative care, we must implement and study strategies such as extended visits, closer partnerships with interpreters as part of the interdisciplinary team, recurrent implicit bias training, and structural changes to increase workforce diversification by discipline, race-ethnicity, and language to minimize disparities in the quality of outpatient palliative care. A collaborative like the PCQN provides an ideal setting in which to conduct quality improvement projects that can evaluate the impact of strategies to address these disparities.

## Figures and Tables

**Table 1:** Characteristics of Patients Seen for a First Visit with Outpatient Palliative Care.

Characteristic	Overall (*N* = 2684)	In-Person (*N* = 1904)	Virtual (*N* = 780)	*P*-value
Age, n (%)				<0.001
18–44	261 (9.9)	189 (10.1)	72 (9.5)	
45–64	1054 (40.0)	811 (43.3)	243 (31.9)	
65–79	986 (37.4)	649 (34.6)	337 (44.3)	
80+	333 (12.6)	224 (12.0)	109 (14.3)	
Sex, n (%)				0.053
Female	1271 (47.6)	881 (46.4)	390 (50.5)	
Male	1400 (52.4)	1018 (53.6)	382 (49.5)	
Reason for referral, n (%)				
Pain	1383 (51.5)	1094 (58.4)	289 (40.0)	<0.001
Other symptom management	1277 (47.6)	843 (45.0)	434 (60.1)	<0.001
GOC/ACP	1256 (46.8)	868 (46.4)	388 (53.7)	<0.001
Transition to comfort care	4 (0.2)	2 (0.1)	2 (0.3)	0.31
Support for patient/family	964 (35.9)	656 (35.0)	308 (42.7)	<0.001
Primary diagnosis, n (%)				<0.001
Cancer	1556 (59.0)	1327 (70.2)	229 (30.6)	
Neurologic	389 (14.7)	132 (7.0)	257 (34.3)	
Cardiovascular	164 (6.2)	115 (6.1)	49 (6.5)	
Pulmonary	208 (7.9)	105 (5.6)	103 (13.8)	
Hepatic	84 (3.2)	47 (2.5)	37 (4.9)	
Other	237 (9.0)	163 (8.6)	74 (9.9)	
Race-ethnicity				<0.001
White	1198 (47.1)	785 (43.0)	413 (57.5)	
Hispanic/Latinx	451 (17.7)	331 (18.1)	120 (16.7)	
Black/African American	236 (9.3)	188 (10.3)	48 (6.7)	
Asian	435 (17.1)	348 (19.1)	87 (12.1)	
Native Hawaiian/Pacific Islander	145 (5.7)	136 (7.4)	9 (1.3)	
American Indian/Alaska Native	6 (0.2)	2 (0.1)	4 (0.6)	
Other	74 (2.9)	37 (2.0)	37 (5.1)	
Language				<0.001
English	1986 (76.3)	1362 (73.7)	624 (82.7)	
Spanish	296 (11.4)	226 (12.2)	70 (9.3)	
Chinese	160 (6.1)	131 (7.1)	29 (3.8)	
Other	161 (6.2)	129 (7.0)	32 (4.2)	
Time				<0.001
Pre-COVID (2017–2019)	1825 (68.0)	1627 (85.5)	198 (25.4)	
COVID (2020–2021)	859 (32.0)	277 (14.5)	582 (74.6)	

Missing data: <2% age (*n* = 50), <1% sex (*n* = 13), <2% primary diagnosis (*n* = 46), 5% race-ethnicity (*n* = 139; 4% in-person, 7% virtual), 3% language (*n* = 81).

**Table 2:** Screening for Process Outcomes Overall and by Visit Type.

ProcessOutcome	Overall (*N* = 2684)	In-Person (*N* = 1904)	Virtual (*N* = 780)	*P*-Value
Psychosocial needs, n (%)				0.18
No	716 (26.7)	522 (27.4)	194 (24.9)	
Yes	1968 (73.3)	1382 (72.6)	586 (75.1)	
Spiritual needs, n (%)				0.18
No	1117 (41.6)	777 (40.8)	340 (43.6)	
Yes	1567 (58.4)	1127 (59.2)	440 (56.4)	
GOC/ACP, n (%)				0.001
No	909 (33.9)	681 (35.8)	228 (29.2)	
Yes	1775 (66.1)	1223 (64.2)	552 (70.8)	

**Table 3:** Screening for Process Outcomes by Language Preference (*N* = 2603).^a^

Process Outcome	English (*N* = 1986)	Non-English (*N* = 617)	Spanish (*N* = 296)	Chinese (*N* = 160)	Other^b^ (*N* = 161)	*P*-Value
Psychosocial needs, n (%)						<0.001
No	455 (22.9)	214 (34.7)	116 (39.2)	51 (31.9)	47 (29.2)	
Yes	1531 (77.1)	403 (65.3)	180 (60.8)	109 (68.1)	114 (70.8)	
Spiritual needs, n (%)						<0.001
No	763 (38.4)	299 (48.5)	151 (51.0)	83 (51.9)	65 (40.4)	
Yes	1223 (61.6)	318 (51.5)	145 (49.0)	77 (48.1)	96 (59.6)	
GOC/ACP, n (%)						<0.001
No	593 (29.9)	264 (42.8)	139 (47.0)	66 (41.3)	59 (36.6)	
Yes	1393 (70.1)	353 (57.2)	157 (53.0)	94 (58.7)	102 (63.4)	

a Missing language preference for *N* = 81 (3%).

b Other language refers to any other non-English language.

**Table 4:** Screening for Process Outcomes by Race-Ethnicity (*N* = 2545).^a^

Process Outcome	White (*N* = 1198)	Hispanic/ Latinx (*N* = 451)	Black/African American (*N* = 236)	Asian (*N* = 435)	Other^b^ (*N* = 225)	*P*-Value
Psychosocial needs, n (%)						<0.001
No	253 (21.1)	154 (34.2)	100 (42.4)	101 (23.2)	49 (21.8)	
Yes	945 (78.9)	297 (65.8)	136 (57.6)	334 (76.8)	176 (78.2)	
Spiritual needs, n (%)						<0.001
No	452 (37.7)	210 (46.6)	130 (55.1)	168 (38.6)	78 (34.7)	
Yes	746 (62.3)	241 (53.4)	106 (44.9)	267 (61.4)	147 (65.3)	
GOC/ACP, n (%)						<0.001
No	355 (28.0)	192 (42.6)	123 (52.1)	128 (29.4)	62 (27.6)	
Yes	863 (72.0)	259 (57.4)	113 (47.9)	307 (70.6)	163 (72.4)	

a Missing race-ethnicity data for *N* = 139 (5.5%).

b Other includes Native Hawaiian/Pacific Islander, American Indian/Alaska Native, and all other races and/or ethnicities not included in other categories.

**Table 5:** Results of Multivariable Models of Screening for Process Outcomes by Visit Type.

Predictor Variables	Screening for Psychosocial Needs aOR (95% CI)	Screening for Spiritual Needs aOR (95% CI)	Screening for Goals of Care Needs aOR (95% CI)
Visit type			
In-person	*Reference*	*Reference*	*Reference*
Virtual	0.87 (0.60–1.25)	0.81 (0.57–1.15)	1.05 (0.85–1.31)
Preferred language			
English	*Reference*	*Reference*	*Reference*
Other	**0.47 (0.38–0.58)**	**0.54 (0.47–0.62)**	**0.48 (0.39–0.59)**
Race-ethnicity			
White	*Reference*	*Reference*	*Reference*
Hispanic/Latinx	0.89 (0.66–1.20)	1.00 (0.74–1.36)	0.95 (0.71–1.28)
Black/African American	**0.38 (0.29–0.51)**	**0.51 (0.38–0.69)**	**0.41 (0.34–0.49)**
Asian	1.43 (0.98–2.09)	1.33 (0.98–1.80)	**1.53 (1.06–2.23)**
Other	1.29 (0.86–1.94)	1.38 (0.96–1.99)	1.39 (0.82–2.38)
Age			
18–44	*Reference*	*Reference*	*Reference*
45–64	0.91 (0.66–1.27)	0.87 (0.60–1.27)	0.93 (0.63–1.36)
65–79	0.93 (0.75–1.16)	0.98 (0.73–1.32)	1.06 (0.81–1.37)
80+	0.99 (0.58–1.71)	0.96 (0.66–1.39)	1.48 (0.93–2.36)
Sex			
Male	*Reference*	*Reference*	*Reference*
Female	1.10 (0.85–1.43)	0.99 (0.73–1.34)	0.96 (0.71–1.31)
Primary diagnosis			
Cancer	*Reference*	*Reference*	*Reference*
Neurologic	**2.21 (1.04–4.71)**	1.06 (0.57–1.98)	2.25 (0.98–5.15)
Cardiovascular	0.97 (0.35–2.69)	0.69 (0.30–1.60)	0.85 (0.32–2.26)
Pulmonary	1.27 (0.78–2.06)	1.09 (0.70–1.68)	1.36 (0.94–1.96)
Hepatic	1.03 (0.43–2.48)	1.05 (0.51–2.15)	1.17 (0.41–3.31)
Other	1.18 (0.71–1.95)	0.84 (0.46–1.54)	0.93 (0.50–1.74)

The bold values are statistically significant.

## Key message

In a multisite study of 2684 outpatient palliative care patients, there were no differences in rates of screening for psychosocial, spiritual, or goals of care needs in virtual versus in-person visits. However, patients with preferred languages other than English and those identifying as Black had lower odds of screening.

## Appendix

**Appendix Table 1:** Results of Multivariable Model of Screening for Spiritual Needs by Visit Type Including Positive Interaction Term of Visit Type by Year (2017–2019 = pre-COVID, 2020–2021 = During COVID).

Predictor Variables	Screening for Spiritual Needs aOR (95% CI)
*Pre-COVID*: Visit type	
In-person	*Reference*
Virtual	1.50 (1.03–2.20)
*During COVID*: Visit type	
In-person	*Reference*
Virtual	0.71 (0.39–1.30)
Preferred language	
English	*Reference*
Other	**0.56 (0.49–0.63)**
Race-ethnicity	
White	*Reference*
Hispanic/Latinx	1.00 (0.75–1.35)
Black/African American	**0.53 (0.39–0.71)**
Asian	**1.35 (1.02–1.79)**
Other	1.38 (0.95–2.01)
Age	
18–44	*Reference*
45–64	0.89 (0.62–1.29)
65–79	1.01 (0.77–1.31)
80+	1.00 (0.71–1.41)
Sex	
Male	*Reference*
Female	0.99 (0.74–1.32)
Primary diagnosis	
Cancer	*Reference*
Neurologic	0.97 (0.54–1.73)
Cardiovascular	0.67 (0.31–1.46)
Pulmonary	0.99 (0.70–1.39)
Hepatic	0.97 (0.51–1.81)
Other	0.83 (0.45–1.53)

The bold values are statistically significant.

There was no correlation or collinearity between predictor variables, including no collinearity between language and race-ethnicity.
